# Performance Degradation Assessment of Concrete Beams Based on Acoustic Emission Burst Features and Mahalanobis—Taguchi System

**DOI:** 10.3390/s20123402

**Published:** 2020-06-16

**Authors:** Md Arafat Habib, Akhand Rai, Jong-Myon Kim

**Affiliations:** School of Computer Engineering and Information Technology, University of Ulsan, 93 Daehak-ro, Nam-gu, Ulsan 44610, Korea; akhtab007@gmail.com (M.A.H.); raiakhand@gmail.com (A.R.)

**Keywords:** concrete crack detection, acoustic emission features, Mahalanobis—Taguchi system

## Abstract

Acoustic emission (AE) has been used extensively for structural health monitoring based on the stress waves generated due to evolution of cracks in concrete structures. A major concern while using AE features is that each of them responds differently to the fractures in concrete structures. To tackle this problem, Mahalanobis—Taguchi system (MTS) is utilized, which fuses the AE feature space to provide comprehensive and reliable degradation indicator with a feature selection method to determine useful features. Further, majority of the existing investigations gave little attention to naturally occurring cracks, which are actually more difficult to detect. In this study, a novel degradation indicator (DI) based on AE features and MTS is proposed to indicate the performance degradation in reinforced concrete beams. The experimental results confirm that the MTS can successfully distinguish between healthy and faulty conditions. To alleviate the noise from the DI obtained through MTS, a noise-removal strategy based on Chebyshev inequality is suggested. The results show that the proposed DI based on AE features and MTS is capable of detecting early stage cracks as well as development of damage in concrete beams.

## 1. Introduction

Concrete beams are widely used in construction of buildings, dams, and bridges. Heavy load, aging, disasters like earthquake can cause severe damage to beam in concrete structures. It is crucial to assess the performance deterioration in concrete structures for preventing economic losses and ensuring safe operations. The detection of the fractures is important to examine the damage in concrete structures. This is usually managed through regular visual inspections that is very difficult to perform because of the inaccessible locations where the fractures have taken place. To overcome this problem, many sophisticated monitoring methods have been considered that use direct or indirect measurements from various sensors for diagnosing fractures in concrete structures [1,2]. These condition monitoring methods can be divided into direct and nondirect methods. Direct methods can be of noncontact or contact type depending upon whether the sensors are directly attached to the test specimen/structure. For instance, direct noncontact techniques include ultrasonic propagation imaging [3] and photographic techniques [4,5,6], whereas direct contact techniques incorporate methods like fiber optics [7] and 2D strain sensing sheets [8]. On the other hand, nondirect methods typically employ monitoring systems based on nondestructive (NDT) techniques such as acoustic emission (AE), thermal methods, ultrasonic methods, vibration analysis, etc. for condition monitoring of the concrete structures [9]. NDT can reveal the internal status of the materials and provide real-time information on the condition of the structure. Of all these methods, recently, AE has been proven to be very effective for composite materials like concrete [10,11,12,13,14,15,16,17]. During the fracture process in concrete beams, materials release energy and generate mechanical waves that propagate through the solid. These mechanical waves can be detected by mounting AE transducers on surface of the concrete specimen [1,3,4,7,8,9,10,13] and consequently, the present condition of the concrete structures can be determined. Since pores and interfaces create heterogeneity, it is a complex task to assess the integrity of the concrete structures.

AE is a feasible tool for data-driven real-time monitoring of concrete structures. Waves involved in AE are related to source mechanism that helps to identify the crack from sensor responses. AE is different from other NDT techniques for two reasons. The first is its unique ability to detect the dynamic processes such as crack propagation and debonding in materials associated with the performance degradation of the structures. The second is that the AE signals of the concrete beams under heavy load can effectively characterize and identify cracks in structures. Generally, the AE signals are classified into two types considering the shapes of the waveforms [4]: (1) Burst type: signals having definite starting and ending points and (2) continuous type: waves with no definite ends having variant amplitude and frequencies. The burst type AE signals are acquired from the brittle materials and they are associated with crack propagation characteristically. Consequently, many research works [8,9,10,11,12,13,14,15,16,17,18,19,20,21,22,23] have adopted AE technique for evaluating degradation in concrete structures. These research articles provided effective approaches for crack/fracture diagnosis in concrete beams, but they failed to address certain key issues that are described as follows:

- The previous researches exploited numerous features such as rise time, decay time, amplitude, energy, ringing counts, RA and AF, etc. for detecting and classifying fractures in concrete structures. Among these features, it is difficult to exactly define features having greater sensitivity to the crack growth. It may happen that certain features are capable of detecting fractures but fail to recognize incipient cracks. Similarly, features sensitive to incipient cracks might not be effective in predicting crack severity (fractures) during final failure stages. It is further emphasized that not only the diagnosis of early stage cracks but the assessment of degradation in concrete structures over their full lifetime is also important to prevent catastrophic failures.
- In many cases, machine learning-based classification algorithms necessitate prior knowledge of failure data in order to implement condition monitoring of concrete structures. In real-life scenarios, there is limited access to such datasets which leads to construction of inefficient degradation assessment models.
- The existing AE features exhibit irregular fluctuations, which make it difficult to determine the time-instants of initial crack occurrence precisely. Further, these features may show nonmonotonic behavior with the increase in degradation severity. This can create false alerts resulting in wrong maintenance decisions.

Thus, it becomes a prerequisite to develop an inclusive degradation indicator (DI) that combines the diagnosis capabilities of individual features and contains less noise. A lot of previous works have considered AE features extracted through the flexural test conducted on concrete beams. Most of these works focus on fault classification (minor/intermediate/severe), crack classification (tensile/shear), fracture identification, analyzing load against AE history, etc. Existing literature lacks in building a solid DI for the degradation assessment of the concrete beams over time. To fill this gap and tackle the above challenges, in this paper, Mahalanobis–Taguchi system (MTS) is utilized to fuse the useful AE features and develop the DI [24]. MTS is a diagnosis and forecasting method that combines MD and Taguchi’s robust engineering method in a system with multiple dimensions. Mahalanobis distance (MD) is used to form a multidimensional measurement scale and define Mahalanobis space (MS) [25]. The MS is used as a reference point of the scale having a group of observations that we can consider as normal. Since MD constructs the measurement scale by incorporating single class samples instead of the whole training data, it can solve the class imbalance problems [25]. MD can be calculated using the distances between the center of the MS and the observations that we are evaluating. If an observation has an MD lesser than the threshold, we consider the observation to be normal. On the contrary, if an observation has a larger MD than the threshold, we can consider that to fall under abnormal category. For concrete fracture identification, MD itself can determine fractures provided that we have appropriate features. Even though MD has been used effectively for cluster analysis and damage classification [26,27,28,29,30], it lacks in mechanism to choose good features that can facilitate us with better trend for DI. Therefore, Taguchi’s robust engineering method that uses orthogonal arrays (OAs) and signal-to-noise ratios (SNRs) is combined with MD to obtain useful features. To the best of our knowledge, this simple yet efficient technique has not been well explored in the existing literature covering composite materials like concrete beams. However, the DI developed using MTS may contain noise and it can be hard to assess the performance degradation due to heavily scattered MD points. To eliminate noise and achieve a monotonic DI, a noise-removal (NR) strategy is suggested [31]. The NR strategy uses the Chebyshev’s inequality to define threshold limits for the beginning of degradation and subsequently, modifies the existing MTS plot to obtain the final DI. Chebyshev’s inequality has been used to detect outliers in data distributions and can be used to monitor changes in the degradation state [32,33]. Overall, the main contributions of the paper are summarized as follows:

- A novel DI showing the time history of the performance degradation of concrete beams is proposed. The DI allows us to observe the development of concrete degradation, beginning from the crack formation till absolute failure of the beams. The DI encompasses wide range of AE features merged by MTS classifier for reflecting the concrete health condition based on the time history of flexural bending test conducted on it. The DI does not require any prior knowledge of failure data for the purpose of degradation assessment.
- A noise-removal (NR) strategy based on Chebyshev inequality is suggested to process the MD in order to obtain smooth and monotonic DI that tends to increase with the growth in degradation severity.

The remainder of this paper is organized as follows. In Section 2, related works are discussed in detail. Section 3 presents the theoretical details of AE features and MTS. Section 4 provides the details of proposed methodology, including the concrete test system for AE data acquisition and DI development. Experimental results and discussions are provided in Section 5. Finally, the paper is concluded in Section 6.

## 2. Related Works

Carpinteri et al. [8] presented a study on numerical modeling accompanied by in-situ monitoring for concrete beams. Yu et al. [10] monitored slow dynamics of microcracks in polymer concrete (PC) beams using AE. The AE data was clustered using k-means clustering algorithm to classify damage states and characterize microdamage mechanisms. Das et al. [11] designed a framework based on hand-crafted features like rise-to-amplitude (RA) and average frequency (AF) that were clustered using an unsupervised clustering algorithm and further separated by a support vector machine (SVM) hyperplane to classify damage conditions. Aggelis et al. [12] investigated the possibility of having feedback from digital image correlation to AE outputs for estimating the fracture behavior of hybrid textile-reinforced concrete beams. Aggelis et al. [13] conducted fracture tests on different types of concrete beams including plain concrete and steel fiber-reinforced concrete with variant coatings, water content ratio, fiber contents, and shapes. Simple cracking modes were characterized and validated based on the AE parameters like AF and RA. Banjara et al. [14] focused on the damage progression in reinforced concrete beams using AE signals. The AE parameters such as AE energy, AE counts, hits, rise time, and amplitude were found effective to detect crack initiation and classify crack types like shear or tensile cracks. Also, the crack initiation could also be detected using the same method. Tsangouri et al. [15] suggested that it is not possible to characterize complex fractures in concrete beams by just observing the changes in mechanical properties, and therefore, AE was used to study the concrete degradation process. The damage-initiation process was determined based on the distribution of cumulative AE hits under increasing load. Kaiser and Felicity effects were considered too. Severity assessment of the damage was done by load/calm ratios. Yue et al. [16] used AE to detect the fracture mode and microcrack initiation in concrete beams. The strain energy release and macrocrack formation was also identified using AE. Chen et al. [17] studied the effect of loading rate on concrete fracture behavior using AE technique. It was found that the rigidity of concrete has profound effects on loading rate. Cumulative AE hits and ringing count were used to detect the initiation of the concrete boundary effect as well as severity of cracks. ElBatanouny et al. [18] utilized AE to map crack growth and identify areas of prominent damage activity leading to the failure of scaled concrete beams made up of glass fiber-reinforced polymer with no shear reinforcement. Their technique successfully distinguished between flexural and shear cracks. Ranjith et al. [19] suggested a long-term AE monitoring technique to diagnose the microcracking events in concrete beams. AE parameters like RA and AF were used to detect crack types and location. Ohno and Ohtsu [20] utilized same parameters, i.e., RA and AF to detect cracks in concrete beams. Yun et al. [21] presented an AE-enabled monitoring system for carbon fiber reinforced polymer concrete. Sagar et al. [22] collected AE data using incremental cyclic loading and exploited AE energy as a key parameter to identify damage in concrete beams. Huang et al. [23] utilized bandwidth, frequency, sensitivity of the embedded AE sensors, etc. for health monitoring of the concrete beams. Study conducted in [23] showed that AE sensors had broad frequency bandwidth and were quite sensitive to crack propagation.

Khatir et al. proposed a method for crack detection by improving the training process of artificial neural network (ANN) parameters by adopting Jaya algorithm [34]. Results were found to be accurate and could successfully predict the potential damage after increasing the regression and controlling the crack propagation. Study conducted in [35] investigated the influence of the beam energy and ion dose of the helium-focused ion beam (He-FIB) on processing damage. The proposed method can perfectly predict the amorphous damage profile induced by He-FIB. Tiachacht et al. proposed a new method to identify and quantify damage in two- and three-dimensional structures [36]. The proposed methodology was investigated numerically through finite element method and MATLAB program. It was effective to estimate the severity of structural damage. Some other works based on inverse problem, optimization techniques, and ANN in the field of fracture and damaged identification were carried out by [37,38,39,40,41,42].

Ebrahimkhanlou et al. [43] have used nonlinear dimensionality reduction, k-means clustering, and hidden Markov models to recognize AE patterns for containment structures. These structures may suffer from hidden declamation cracks, and it is very hard to detect them. Validation was done using large-scale concrete walls, and early detection of declamation was successfully performed. Ebrahimkhanlou et al. used AE techniques to monitor a large-scale curved, post-tensioned concrete wall under monotonically increasing prestressing loads [44]. A network of AE sensors was built mounted on the outer surface of the wall to determine patterns in AE signals. Clustering of the AE signals were achieved using k-means clustering algorithm. Sequence of those signals were modeled using the Markov model. Ebrahimkhanlou et al. further proposed an AE-based method to monitor delamination for post-tensioned concrete containment structures in [45]. AE data were analyzed using both time-driven and hit-driven features extracted from AE. In [46], an AE monitoring approach was proposed for old steel bridges strengthened with post installed shear connectors. The proposed method leverages the difference in AEs from shear connectors before and after they engage in shear transfer.

## 3. Technical Background

### 3.1. Acoustic Emission Burst (AEB) Features

**Figure 1:**
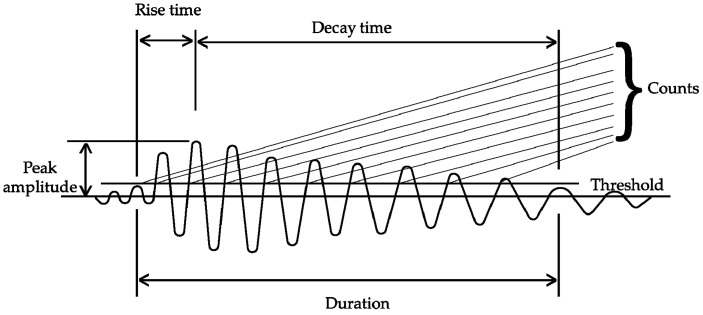
Definition of the acoustic emission (AE) features.

AE signals obtained from the concrete structures have special parameters/features that can describe AE events. The AE features considered in this paper are the ones used to characterize AE bursts. Features we have considered are peak amplitude, rise time, decay time, AE counts, and AE energy. These parameters will be collectively referred as AEB features in our study. The AEB features are considered to be good measures for detecting cracks and possess good relation to the concrete structural health condition. Figure 1 describes the AEB features, which are further defined as follows [47,48]:

**Peak amplitude:** The voltage that is measured as the highest one in AE waveform is regarded as the peak amplitude.

**Rise time:** It is defined as the time interval between the triggering time and the peak of the AE signal.

**Decay time**: Decay time is opposite to rise time. It is defined as the time when the amplitude decreases from the peak value to the lowest within the set threshold.

**Counts:** AE count is the number of times when the signal crosses the threshold. This feature is also used to measure the strength of the hits and AE activity.

**Energy:** Finally, AE energy is the area measured within the rectified signal envelope.

### 3.2. Mahalanobis–Taguchi System (MTS)-Based Classifier

The first step to implement MTS is to calculate the MDs of the normal observations. In order to do this, features that denote normal condition are defined first. After that, data of all the features presenting normal condition are collected. MDs of all the normal observations are calculated next. Let as assume the constructed feature set be:

(1) $$F_{m\times n}={\left[f_{uv}\right]}_{m\times n},$$

where $f_{uv}$ represents the *u*th observation of the *v*th reference, *m* presents the total number of observations, and the total number of features are denoted by *n*. By definition, it is necessary for every single variable to contribute equally to find out MD. Therefore, the observations in *F* has to be normalized. It can be normalized using the following equation:

(2) $$N_{uv}=\left(f_{uv}-{\bar{f}}_{v}\right)/\sigma_{v},$$

where $N_{uv}$ represents the normalized test feature and *σ* is the standard deviation. ${\bar{f}}_{v}$ and *σ* can be calculated as:

(3) $${\bar{f}}_{v}=\frac{1}{a\sum_{u=1}^{a}f_{uv}},$$

(4) $$\sigma =\sqrt{\sum_{u=1}^{a}{\left(f_{uv}-{\bar{f}}_{v}\right)}^{2}/\left(a-1\right)},$$

where *a* is the number of healthy observations in the dataset. Finally, MD is calculated using the following equation considering the normalized test feature:

(5) $$MD\left(u\right)=\sqrt{\frac{1}{q}}N_{uv}C^{-1}N_{uv}^{T},$$

where *C* is the correlation matrix for the feature we normalize. One critical issue that arises while calculating MDs is multicollinearity. It occurs if there are profound correlations among the features. It leads to an approximate singular correlation matrix that imposes difficulty to compute MDs. We have used Gram–Schmidt orthogonalization process (GSP) [49,50,51] to tackle this issue of multicollinearity. Thus, MDs can be computed using GSP and the computation process is as follows:

Let us assume $Q_{1},Q_{2},\ldots ,Q_{y}$ to be linearly independent vectors. For these vectors, there exist mutually orthogonal vectors that can be represented as $O_{1},O_{2},\ldots ,O_{y}$. These mutually orthogonal vectors have same linear span. The Gram–Schmidt vectors considering the assumed variables are:

(6) $$\{\begin{array}{c} O_{1}=Q_{1},\\ O_{2}=Q_{2}-\frac{Q_{2}^{T}O_{1}}{O_{1}^{T}O_{1}}O_{1}\\ .\\ .\\ .\\ O_{y}=Q_{y}-\frac{Q_{2}^{T}O_{1}}{O_{1}^{T}O_{1}}O_{1}-\ldots -\frac{Q_{y}^{T}O_{y-1}}{O_{y-1}^{T}O_{y-1}}O_{y-1} \end{array}$$

Assuming that *j* refers to a particular feature we are interested in, we can write: $Q_{j}={\left[q_{1j},q_{1j},\ldots ,q_{nj}\right]}^{T}$ and $O_{j}={\left[o_{1j},o_{1j},\ldots ,o_{nj}\right]}^{T}$, where j=1,2,3,…,y. The MD for the *k*_th_ observation of the *j*_th_ feature can be calculated using the following equation:

(7) $$MD_{k}^{2}=\frac{1}{y}\left(\frac{o_{1j}^{2}}{{\hat{\sigma }}_{1}^{2}}+\frac{o_{2j}^{2}}{{\hat{\sigma }}_{2}^{2}}+\ldots +\frac{o_{ky}^{2}}{{\hat{\sigma }}_{y}^{2}}\right),$$

where k=1,2,3,…,n and $\hat{\sigma }$ represents the standard deviation of the *j*_th_ feature.

In the second step, MS is used to calculate the MDs of the abnormal group. MS can be denoted as the zero-point, unit distance and is used as the reference point for the measurement scale. For our implementation, abnormal group consists of feature points associated with the crack formation in the concrete beam. Features extracted from the abnormal condition should also be normalized. They are normalized adopting the mean and standard deviation of the corresponding features in the normal group. The correlation matrix that corresponds to the normal group is used to calculate the MDs of the abnormal condition. MDs of the abnormal condition are supposed to have higher values and a sharp rise in the time-history plot can be observed.

The third step involves finding out useful features to feed the system. As mentioned earlier, MD itself is enough to build time-history trend for faulty conditions. The problem is that we cannot assure features that we have used, if all of them are useful. Using too many features some of which are not even worth using can make the computation process slower. Therefore, Taguchi method is used to select the useful features for calculating MDs. Useful features are sorted out using OAs and SNRs. In MTS, useful feature determination is performed using a two-level OA. Level one includes the feature we are dealing with, whereas level two excludes the feature we are interested in. SNRs calculated from the abnormal conditions are used as response column for every single combination of OA. Usually, larger-the-better type SNR is used to sort out useful features. It is calculated using the following equation:

(8) $$SNR=-10\log_{10}\left(\frac{1}{m}\sum_{t=1}^{m}\frac{1}{MD_{t}^{2}}\right),$$

where *m* is the number of the abnormal conditions and *MD_t_* is the MD of the *t*th abnormal condition.

We have to calculate the effective gain of each feature after computing the SNR. ${\bar{SNR}}_{L-1}$ refers to the average SNR of all the runs including the feature and ${\bar{SNR}}_{L-2}$ represents the same excluding the feature we are interested in. Thus, the effective gain of a particular feature can be found using the flowing equation:

(9) $$Gain={\bar{SNR}}_{L-1}-{\bar{SNR}}_{L-2}.$$

If the gain we calculate is positive, we consider the feature for our system. Otherwise, we exclude the feature while calculating the degradation indicator associated with our concrete crack detection. As the feature points of the increasing timeline would eventually correspond to the abnormal data, we will figure out the potted line is sharply rising giving us insight that crack formation has taken place.

## 4. Methodology

The proposed methodology can be divided into two main steps. The first one is the acquisition of raw AE data mining the degradation in concrete. For this purpose, an experimental setup was developed to conduct three-point bending test on concrete beams in the presence of an external load. The second one is building an effective DI by utilizing AEB features and MTS classifier that will help us to assess the performance degradation in concrete beams. These steps have been described into the upcoming sections.

### 4.1. Experimental Test Bed and Data Acquisition

The test bed was developed in our laboratory for acquiring AE data. Usually, flexural test is used to evaluate the tensile strength of concrete beam and measure its ability to endure failure caused due to bending. This test is carried out using either three-point bending or center-point bending. Three-point bending is implemented in such a way that half of the total load is applied at every one-third of the concrete beam span length. Thus, the concrete beam experiences maximum stress over its central one-third portion. On the contrary, in center point bending, the entire load is applied at the center of the concrete beam. According to American Society for Testing and Materials (ASTM), the length of the concrete beam should be at least three times greater than its depth [52]. In present case, the tested concrete specimen had a depth of 300 mm. We have fixed the span length to 2400 mm for the experiment. For such large length, it is necessary to use three-point bending test to ensure uniform spread of cracks in the beam. As such, experiments were conducted using three-point bending test on reinforced concrete beams. Proportion of the materials used to build the concrete beams for our experiments are described in Table 1.

**Figure 2:**
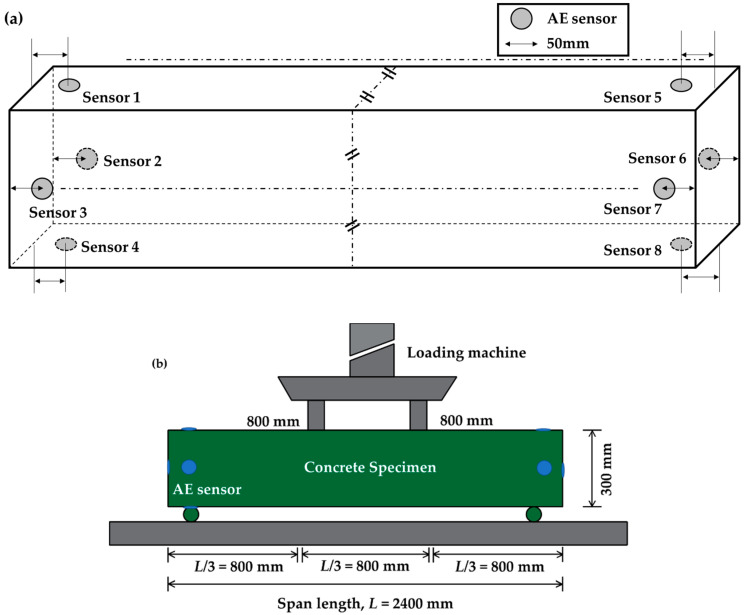
Schematic diagrams of the setup: (**a**) placement of the sensors and (**b**) the flexural test for the concrete beam (three-point bending).

**Figure 3:**
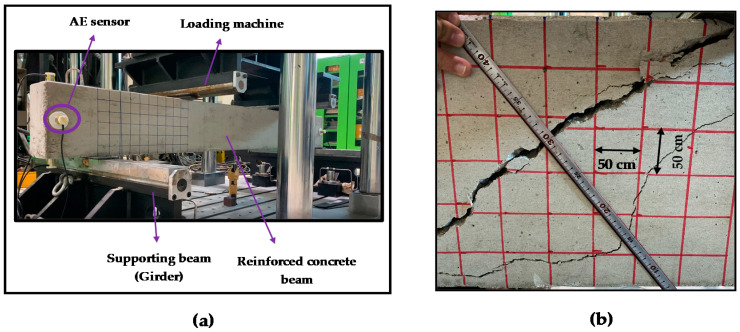
Actual experimental set-up of the three-point bending test for concrete beam: (**a**) whole setup and (**b**) measuring scale used to measure the cracks.

**Figure 4:**
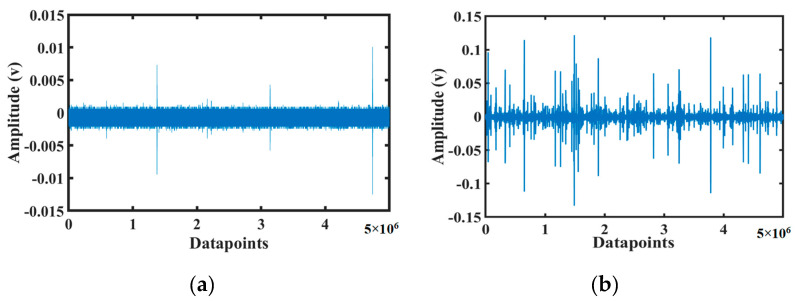
Collected noisy AE signals from the three-point bending test: (**a**) normal condition and (**b**) abnormal condition.

Figure 2 and Figure 3 depict the concrete beam test bed designed for collecting AE signals. Figure 2 presents the schematic diagram for the placement of the sensors in the concrete beam and the three-point bending test. Figure 3 presents the actual setup developed in the laboratory. The length and depth of the concrete beam was 2400 and 300 mm, respectively. A gradually increasing external load was applied for a certain time to initiate cracks. The loads were applied through two locations lying at a distance of 800 mm from each other. The load velocity was kept at 2 mm/s. Different types of sensors were tested for data acquisition. Three types of sensors were used in the experimental setup: R3I [53], WD [54], and R15I [55]. For each sensor type, the experiment was conducted three times in the same condition for reproducibility test. Among these three sensors, we have chosen to use R3I sensor. R3I sensor was chosen due to its better performance. This sensor is built for structural health monitoring of concretes and geological structures [53]. R3I sensors specialize in rejecting high-acoustic background noise and work fine when placed in proximity. A major concern while using AE sensors is the attenuation of stress waves before it reaches the sensors. Before carrying out the fracture tests, pencil lead break (PLB) tests were implemented at appropriate locations to check the responsiveness of different sensors towards AE waves. It was observed that among the three sensors, the R3I sensor possessed better sensitivity towards the AE signals generated by PLB tests. As such, the R3I sensor was utilized to acquire the AE signals from the concrete beam specimens. Eight sensors in each experiment were placed in the concrete beam. As concrete beams are three-dimensional rectangular entity with 6 surfaces, we have tried to cover all the surfaces using eight sensors in total. The AE sensors were fixed to the surface of concrete specimen by using mounting tapes and glue gels. Locations were put as far as possible from the loading machine, mostly at the end points of the concrete so that the cracks/fractures formed due to load do not interrupt the position of the sensors. We have followed two strategies while placing the sensors. The first one is to cover all the six surfaces of the concrete beams and another is the interruption avoidance to the sensors due to crack formation. To obtain intrinsic information, the AE signals were collected at a sampling rate of 10 MHz, and the total signal length was taken as approximately 17.25 min. The R3I sensors contained an integrated filter and preamplifier with gain of 40 db. The sensors have an operating frequency range of 10–40 kHz. No prior AE threshold was set while conducting the experiments. Instead of this, an adaptive threshold level depending upon the maximum amplitude of acquired AE signals was utilized. This was done to minimize any possible information loss in an AE signal because of large threshold setting caused by ambiguous judgement of background noise levels. A constant threshold level for all AE signals may create false triggers for bursts and lead to inaccurate calculation of AEB features. As such, the threshold level was set empirically to 10% of the peak amplitude of an AE signal. Figure 4 portrays the AE signals for two different conditions of the concrete beam. The first condition was the normal mode when there was no crack/fracture formation. The second condition denotes the state of abnormal condition of the concrete beam caused by an external load resulting in fracture formation.

**Figure 5:**
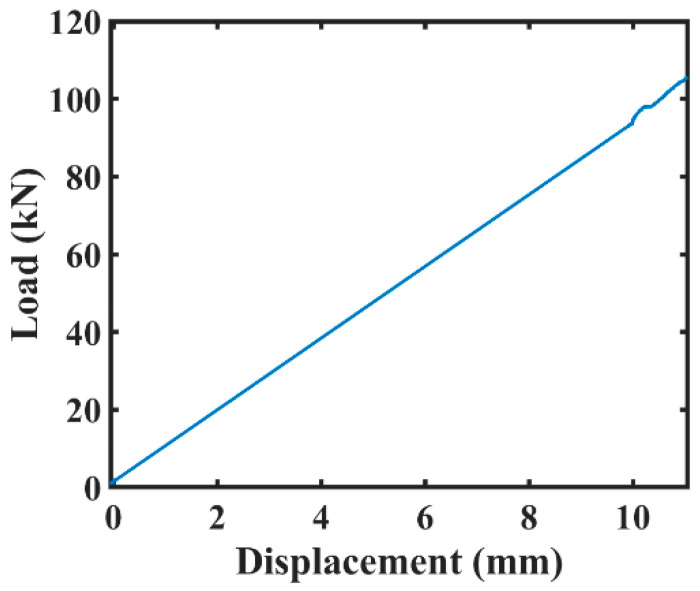
Load vs. displacement plot.

**Figure 6:**
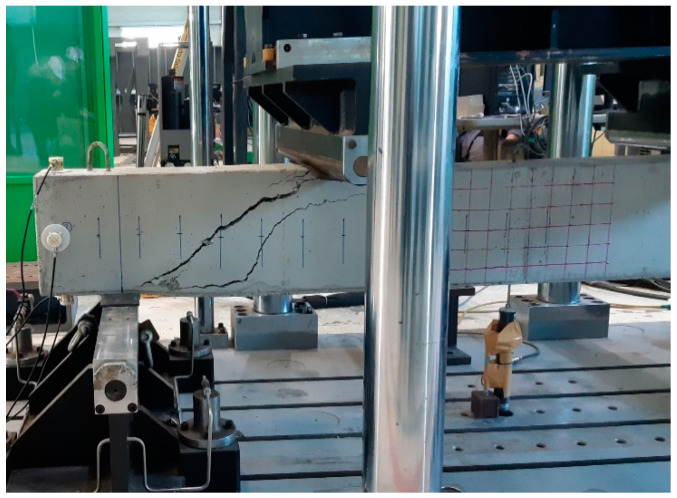
Severe fracture that took place after applying a gradually increasing load.

After the load was applied gradually, cracks were formed in the concrete specimen. The data of the applied load and corresponding displacement was carefully recorded. This helps to validate the output results by comparing the load and displacement curves with the AE features acquired during the failure history of concrete specimen. Figure 5 presents the load vs. displacement graph that shows us the increase of displacement as the load was increased through time. The concrete beam’s in-plane displacement was measured at its mid-span using linear variable differential transformer (LVDT). The LVDT was fixed to the bottom surface of concrete beam specimen. Figure 6 portrays the concrete specimen after the failure has occurred due to gradually applied load. Table 2 describes the specifications of the experiments conducted in the laboratory. 

### 4.2. Proposed Technique for DI Development Using MTS and NR Strategy

The proposed DI for performance degradation assessment of concrete structures was constructed into the following steps:

*Step* *1.* The AE signals were collected in real time from the experimental setup designed for implementing three-point bending tests on the reinforced concrete beam.
*Step* *2.* The AEB features discussed in Section 3.1 were extracted from the raw AE signals.
*Step* *3.* MDs of the normal observations were calculated. For this, features that denote normal condition were defined first. After that, data of all the features presenting normal condition were collected. MDs of all the normal observations were calculated next.
*Step* *4.* MS was used to calculate the MDs of the abnormal group. For our implementation, abnormal group consisted of feature points associated with the fracture formation in the concrete beam. Features extracted from the abnormal condition were normalized. They were normalized adopting the mean and standard deviation of the corresponding features in the normal group. The correlation matrix that corresponded to the normal group was used to calculate the MDs of the abnormal condition.
*Step* *5.* Taguchi method was used to select the useful features for calculating the MDs for the final plot. Useful features were sorted out using OAs and SNRs. The SNRs obtained from the abnormal MDs representing fractures in the concrete were used as the response column for each combination of OA.
*Step* *6.* The MD was processed using the NR strategy to remove noise and obtain a monotonic DI. The suggested NR strategy was implemented as follows:
  First, the NR approach utilizes Chebyshev inequality to define threshold limits for declaring the beginning of degradation in concrete beams. Mathematically, the Chebyshev inequality is given as $P\left(\left|N-\mu_{n}\right|>\varepsilon \right)\leq \sigma_{n}/\varepsilon_{n}^{2}$, where *N* denotes the MD values estimated for normal or healthy conditions, *μ_n_* and *σ_n_* denote the mean and standard deviation, respectively, of dataset *N*, and *P* denotes the probability of the elements in data *N* deviating *ε_n_* away from the mean *μ_n_*. Let us assume that the threshold set for incipient crack detection is *μ_n_* + *ε_n_* where *ε_n_* = 3*σ_n_*, then the Chebyshev inequality states that no more than 89% (=1 − 1/3^2^) of the values in *N* are more than 3 standard deviations away from the mean value. Thus, an accuracy of 89% can be achieved in discriminating the faulty MD values from the healthy ones.
  Second, the MD values below the threshold are set to zero and the MD deviations above the threshold are added successively over time to form the desired DI. Let *MD_i_*, (i=1,2,…,L) be the MD time-series to be processed for removing the noise, then the final DI is obtained as follows:
  (10) $$DI\left(i\right)=DI\left(i-1\right)+DI_{t}\left(i\right),\;\mathrm{where}\;DI_{t}\left(i\right)=MD\left(i\right)-\left(\mu_{n}+3\sigma_{n}\right).$$
  (11) $$DI_{t}\left(i\right)=0,\;\mathrm{if}\;MD\left(i\right)\leq \mu_{n}+3\sigma_{n}.$$

**Figure 7:**
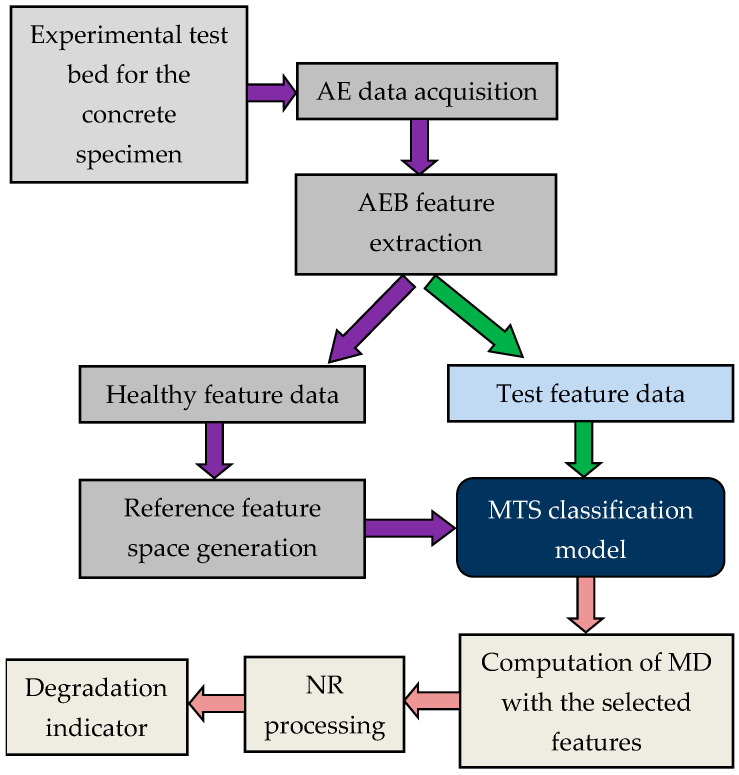
Proposed framework for building degradation indicator (DI) for degradation assessment of concrete beams.

Figure 7 further provides a layout of the above discussed steps for estimating the performance degradation in concrete beams.

## 5. Result Analysis

**Figure 8:**
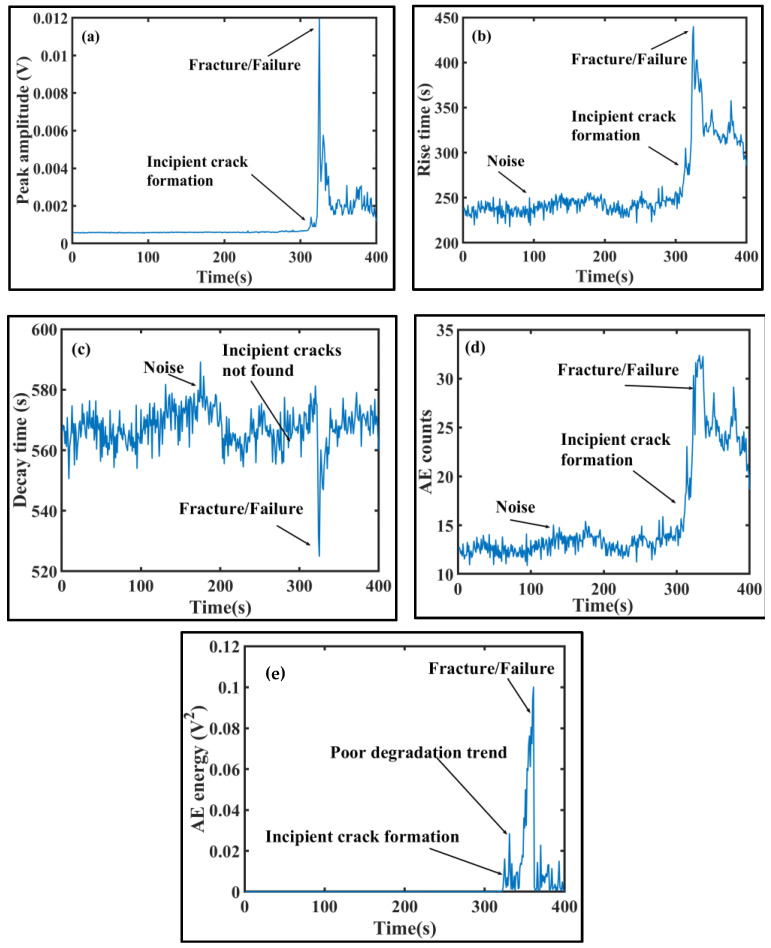
Time history of acoustic emission burst AEB features over the entire life of concrete beam: (**a**) peak amplitude, (**b**) rise time (**c**) decay time, (**d**) AE counts, and (**e**) AE energy.

The analyses in this paper were carried out using the MATLAB software. The proposed technique discussed in Section 4.2 is applied to validate its potential in predicting degradation of concrete beam specimen. First, the AEB features are extracted from the acquired AE signals. Trends of five AEB features (peak amplitude, rise time, decay time, AE counts, and AE energy) over the entire failure history of concrete specimen are displayed in Figure 8a–e. An AE signal sample can contain many bursts and therefore, a time-window of 1000 data points is taken to compute AEB features. The threshold is set to 10% of the maximum value, and the features calculated for different windows are averaged. Figure 8a, shows very high increase as the crack becomes critical leading to fractures. In Figure 8b, a sharp increase in the time-history curve is observed as the cracks are formed. Figure 8c shows a sharp decline in decay time curve after the development of cracks. In Figure 8d, the time history of AE counts can distinguish between the normal and crack mode since higher AE counts are observed in presence of cracks. Figure 8e shows that high energy values are reported as cracks develop in the concrete beam. Overall, it is observed that the AEB features like peak amplitude, rise time, counts, and AE energy first show a slow growth indicating the occurrence of cracks. Then, they rise sharply after certain time signifying the crack size has drastically enlarged leading to fractures and the concrete beam has failed completely onward. For decay time, the trend is not very much clear unless the failure stage is reached where a sudden decrease in decay time is observed. Also, the crack propagation is rapid due to brittle nature of concrete. Consequently, there is less time between crack initiation, fracture, and critical failure. After the concrete beam fails totally, it will not be able to sustain the applied load and no valid AE signals are recorded. Hence, the AEB features show irregular variations and inconsistent trends after attaining maximum value at fracture. It can be realized that different features show different behavior in terms of spikes when there is a crack, and also the oscillation density is different around the moment of crack initiation. For instance, the time-history curve of peak amplitude shows minimal oscillations, whereas the time-history curves of remaining features oscillate heavily and show noisy behavior. Figure 8a–e further show that these features possess different sensitivities to the evolution of cracks and change by different magnitudes as cracks propagate to fracture. In addition, features like AE energy increase when cracks appear but immediately decrease to lower levels and follow a nonmonotonic trend. However, the specific reason for obtaining the mentioned features is to get good enough variation in data for the faulty conditions with respect to the healthy ones. Table 3 presents the mean values of individual AEB features for a time-window of 100 s considered under different health states of concrete specimen. It can be clearly seen that there are significant changes in feature values as the concrete condition changes from normal to incipient (crack) and then to severe (fracture). This is necessary to build up the Mahalanobis feature space for discriminating the faulty conditions from the normal conditions and achieve accurate performance degradation assessment of concrete specimen. After the features have been extracted, the reference feature space for MTS classifier is constructed. For this purpose, the concrete specimen is assumed to be in healthy conditions for the initial 200 s and as such, the first 200 feature vectors form the reference data for training MTS classifier. The remaining feature vectors are tested against the reference feature set, and the corresponding MD is estimated. To choose the better features for calculating our final MD, we have used Taguchi’s method. Table 4 shows the response table for the SNRs of all the features in both levels. Using Equation (9), we can calculate the gain for each feature, and it can be observed that AE counts, decay time, and rise time have positive gains and are selected for plotting the MD that correspond to the concrete’s failure history.

**Figure 9:**
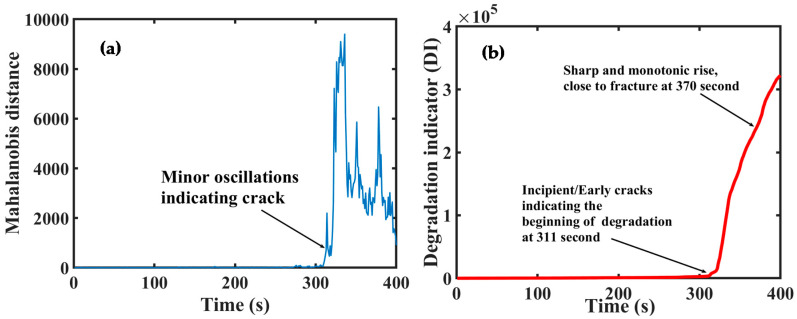
Time-history of Mahalanobis distance (MD) and DI over the entire lifetime of concrete beam: (**a**) fusing all the selected features in MD and (**b**) DI using MD and noise-removal (NR) approach.

A plot of the MTS for the concrete’s failure history is presented in Figure 9a. The plot combines together all the fault information from each AE feature selected via MTS and reflects the performance degradation of the concrete beam in a more comprehensive manner. It can be observed from Figure 9a that the plot contains less noise and presents a clearer degradation trend as compared to the individual AEB features. However, the MTS curve still contains minor oscillations before it begins to grow fully, which in turn can lead to undesirable maintenance alarms. To achieve further accuracy, it is finally processed using the NR technique to obtain the recommended DI plotted in Figure 9b. Figure 9b shows that the DI is completely free of noise which makes it easier to determine the moment of early degradation more conclusively. In addition, the DI increases monotonically as the concrete specimen progresses towards failure. This gives an exact impression of the increase in degradation severity. The DI starts growing at a time-step of 311 s indicating that the incipient cracks have developed and the degradation of concrete has begun. The DI continues to increase rapidly till complete failure of the concrete beam has taken place. Thus, the results confirm that the proposed technique has a good potential in predicting the performance degradation in concretes with higher accuracy and less chances of incorrect maintenance decisions.

## 6. Conclusions

The paper proposes an approach combining AEB features and MTS to build a novel DI for assessing performance degradation of concrete beams. First, the raw AE signals are acquired from the test bed where the concrete beam is subjected to a three-point bending test. Second, five features, namely, peak amplitude, rise time, decay time, AE counts, and AE energy are extracted to form the feature space. These features are supplied to the MD classifier which distinguishes the damage conditions from the healthy ones. Third, the usefulness of the extracted features is determined through Taguchi’s robust engineering method using OAs and SNR. The SNRs obtained from the abnormal MDs representing fractures in the concrete beam are used as the response for each combination of OA. Rise time, decay time, and AE counts are selected in the process as better features due to their positive gain. Finally, MDs are calculated with the sorted-out features and the final MD plot is filtered to remove noise and build a reliable DI for the concrete beam. The new DI can effectively trend the degradation in concrete beam over its time history. The DI could successfully highlight the time when the premature cracks occur and also, the time when failure took place. The proposed method is industry friendly in the sense that it depends only on the healthy data and does not require any previous information of the failure data. Our upcoming work will be to use hybrid feature space and machine learning algorithms, both supervised and unsupervised, for crack detection and crack type classification in real concrete structures.

## Figures and Tables

**Table 1:** Elements of the 24 MPa concrete beams used in the experimental setup.

Elements	Amount
Cement	280 kg/m^3^
Water	202 kg/m^3^
Sand	777 kg/m^3^
Coarse aggregate	988 kg/m^3^
Steel reinforcement: Korean Standard Reinforced Steel Bar, D16 (SD400)	4% of total cross-sectional area of a beam

**Table 2:** Specifications of the experiments.

Specification	Value
Number of sensors per experiment	8
Types of sensors	R3I, WD, R15I AE sensors
Selected sensor	R3I
Total number of concrete beams used for all the tests	10
Minimum signal acquisition time	9 min
Maximum signal acquisition time	17.25 min
Type of concrete used	24 MPa
Steel bars used for reinforcement	Korean Standard Reinforced Steel Bar, D16 (SD400)
Initial load	1.03 kN
Final load	107.6 kN
Load velocity	2 mm/s
Type of displacement	In-plane
Location from which the displacement was measured	Mid-span

**Table 3:** Mean values of different acoustic emission (AE) features in every 100 s of the healthy (normal mode) and faulty condition (fracture mode).

AEB Feature	Mean of Feature Values for 100 s (Normal Stage)	Mean of Feature Values for 100 s (Incipient Crack Stage)	Mean of Feature Values for 100 s (Failure Stage)
Peak amplitude	5.7462 × 10^−4^ V	6.1033 × 10^−4^ V	0.0022 V
Rise time	235.3067 s	241.1678 s	319.5551 s
Decay time	565.3067 s	565.0535 s	567.0314 s
AE counts	12.4312 (unitless)	13.157	23.6754
AE energy	1.5437 × 10^−6^ V^2^	6.080 × 10^−6^ V^2^	0.012345 V^2^

**Table 4:** Response table for signal-to-noise ratios (SNRS) to sort out useful features.

AEB Feature	Level 1	Level 2	Gain
Peak amplitude	15.02	15.73	−0.71
Rise time	16.42	14.33	2.09
Decay time	16.86	13.89	2.97
AE counts	16.13	14.62	1.51
AE energy	14.00	16.75	−2.75
